# Uterus‐preserving surgery for placenta accreta spectrum: An expert, illustrated review of techniques and its outcomes

**DOI:** 10.1111/aogs.70291

**Published:** 2026-07-13

**Authors:** Pedro Viana Pinto, Albaro José Nieto‐Calvache, Rafael José Vieira, Abdalla Mousa, Hooman Soleymani majd, Rozi Aditya Aryananda, José Miguel Palacios‐Jaraquemada

**Affiliations:** ^1^ Serviço de Ginecologia ULS São João Porto Portugal; ^2^ Departamento de Anatomia Faculdade de Medicina da Universidade do Porto Porto Portugal; ^3^ Departamento de Ginecología y Obstetricia Fundación Valle del Lili Cali Colombia; ^4^ Universidad ICESI Facultad de Ciencias de la Salud Cali Colombia; ^5^ Department of Community Medicine, Information and Health Decision Sciences (MEDCIDS), Faculty of Medicine University of Porto Porto Portugal; ^6^ Centre for Health Technology and Services Research, Health Research Network (CINTESIS@RISE), Faculty of Medicine University of Porto Porto Portugal; ^7^ Department of Obstetrics and Gynaecology, Faculty of Medicine, Kasr Alainy Hospital Cairo University Cairo Egypt; ^8^ Oxford University Hospitals NHS Foundation Trust, Department of Gynaecology Oncology Churchill Hospital Oxford UK; ^9^ Nuffield Department of Women's and Reproductive Health University of Oxford Oxford UK; ^10^ Maternal–Fetal Medicine Division, Obstetrics & Gynecology Department, Faculty of Medicine Universitas Airlangga Dr. Soetomo General Academic Hospital Surabaya Indonesia; ^11^ Department of Obstetrics & Gynecology Erasmus University Medical Center Rotterdam The Netherlands; ^12^ Hospital Universitario CEMIC Buenos Aires Argentina

**Keywords:** cesarean hysterectomy, conservative management, myometrial resection, placenta accreta spectrum, placenta in situ

## Abstract

Placenta accreta spectrum (PAS) disorders are associated with substantial maternal morbidity and remain one of the most challenging conditions in modern obstetrics. Although cesarean hysterectomy with the placenta left in situ is widely regarded as the standard treatment, uterus‐preserving surgical strategies have emerged as viable alternatives in carefully selected cases managed in specialized centers. Building on our recently published systematic review and meta‐analysis, this expert‐illustrated review provides a comprehensive and critical appraisal of conservative surgical approaches for PAS, with a particular focus on operative techniques, patient selection, and short‐ and long‐term outcomes. We clearly differentiate two fundamentally distinct conservative strategies: myometrial resection techniques and leaving the placenta in situ. For each approach, we synthesize current evidence, describe procedural nuances, highlight technical prerequisites, and discuss expected intraoperative and delayed complications. The most commonly performed myometrial resection techniques—including one‐step conservative surgery, the Triple‐P procedure, and the Kasr Alainy technique—are detailed step‐by‐step and illustrated to emphasize key surgical principles. Expectant management with the placenta left in situ is also reviewed, with particular attention to indications, postoperative surveillance, delayed morbidity, and fertility outcomes. Data from 50 studies encompassing 2659 patients undergoing myometrial resection and 552 managed with the placenta left in situ are integrated to contextualize reported outcomes and failure rates. This expert review aims to support clinical decision‐making by clarifying the indications, limitations, and risks of conservative PAS management. We argue that uterus‐preserving strategies can be safely and effectively implemented in selected patients when performed by experienced multidisciplinary teams, while emphasizing that readiness to convert to hysterectomy remains essential. By distinguishing techniques and aligning them with patient‐specific factors, this review provides practical guidance for maternal–fetal medicine specialists managing PAS in contemporary clinical practice.

## Graphical abstract

Uterus‐preserving strategies for PAS include myometrial resection and leaving placenta in situ, differing in surgical principles, complications, and follow‐up. Myometrial resection offers definitive treatment with lower rates of delayed morbidity, while leaving the placenta in situ minimizes surgical risk but is associated with prolonged follow‐up.

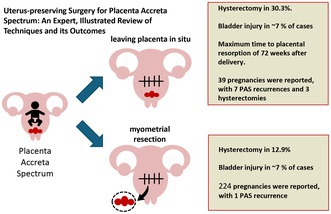

**Abbreviations**
FIGO: International Federation of Obstetrics and Gynecology
ISPAS: International Society for Placenta Accreta Spectrum
OSCS: one‐step conservative surgery
PAS: placenta accreta spectrum

**Key message**

Uterus‐preserving strategies for PAS include myometrial resection and leaving the placenta in situ. Myometrial resection offers definitive treatment with low rates of delayed morbidity, while leaving the placenta in situ minimizes surgical risk but is associated with delayed morbidity.

## 1 INTRODUCTION

Placenta accreta spectrum (PAS) disorders result from abnormal placentation in which chorionic villi abnormally adhere to or penetrate the myometrium through a defect in the decidua.^1^ PAS is associated with substantial maternal morbidity, primarily due to massive hemorrhage and injury to adjacent pelvic organs, including the urinary bladder, ureters, and bowel.^2^

Once considered a rare obstetric complication, PAS has become increasingly prevalent, with recent estimates ranging from 1:533 to 1:730 deliveries.^3^, ^4^ This rise is largely attributed to increasing cesarean delivery rates, particularly when combined with placenta previa.^5^ This is particularly important in countries with high cesarean rates and high fertility rates, which can have a higher number of PAS cases, where cumulative risk across multiple pregnancies is substantial.^6^

Cesarean hysterectomy with the placenta left in situ remains the most widely accepted and commonly performed treatment for PAS worldwide.^7^, ^8^, ^9^, ^10^ When undertaken electively by experienced multidisciplinary teams using standardized surgical protocols, excellent outcomes have been reported.^11^, ^12^ Nevertheless, uterus‐preserving strategies have gained increasing attention over the past two decades. These include myometrial resection techniques and expectant management with the placenta left in situ, developed with the intent of reducing surgical morbidity and, in selected cases, preserving fertility.^13^, ^14^

Evidence supporting conservative approaches remains heterogeneous. In a large multicenter prospective study, uterus‐preserving strategies were shown to be feasible, yet conversion to hysterectomy occurred in nearly half of planned myometrial resections and more than one‐third of planned expectant management cases. Moreover, conservative procedures were unevenly distributed across centers, with relatively few institutions performing them regularly, underscoring the importance of surgical expertise and institutional experience.^15^

Interpretation of the literature is further complicated by inconsistent terminology. Some authors use “conservative management” to describe leaving the placenta in situ, whereas others use it to describe myometrial resection techniques. Several studies combine both approaches in pooled analyses, despite their fundamentally different surgical principles, risk profiles, and expected outcomes.^16^ Both types of conservative treatments have advantages and complications. While a recent systematic review and meta‐analysis^17^ quantitatively assessed outcomes of conservative management, the present expert review aims to provide a practical, illustrated, and technique‐focused synthesis of uterus‐preserving strategies for PAS. We clearly distinguish between myometrial resection and leaving the placenta in situ, detailing operative principles, indications, limitations, and associated morbidity to support informed clinical decision‐making in contemporary PAS management.

## 2 MATERIAL AND METHODS

This expert review builds upon a recently published systematic review and meta‐analysis.^17^ A comprehensive literature search was performed to identify studies describing uterus‐preserving surgical techniques for PAS, using combinations of terms related to PAS and conservative or uterus‐sparing management. Two reviewers independently screened titles, abstracts, and full texts.

The included studies were categorized according to the conservative strategy: myometrial resection techniques or leaving the placenta in situ. Preference was given to studies providing detailed operative descriptions and larger patient cohorts. Extracted outcomes included estimated blood loss, transfusion requirements, injury to adjacent pelvic organs, intensive care unit admission, need for immediate or delayed hysterectomy, infectious morbidity, and fertility outcomes. Given the narrative and expert focus of this review, formal quality assessment tools were not applied.

## 3 RESULTS

The primary analysis included 35 studies describing myometrial resection techniques (2659 patients) and 15 studies describing expectant management with the placenta left in situ (552 patients). Most studies were retrospective case series, with six prospective studies available. Study characteristics and outcomes are summarized in Tables 1, 2, 3.

**TABLE 1:** Summary of myometrial resection techniques reported for PAS.

Author/year	Type of study	Number of patients	Gestational age at delivery (mean)	Blood loss (mL)	Erythrocyte transfusion (units)	Urological interventions (%)	Hysterectomy (%)	Other outcomes (%)
One‐step conservative surgery								
Palacios‐Jaraquemada 2004^54^	Case series	68	Not reported	Not reported	S1–1.6 (0–5)*	Ureteral injury 2 (2.9)	S1–2(4.4)	
					S2–6.9 (5–10)*	Vesical fistula 1 (1.5)	S2–16 (73)	
Palacios‐Jaraquemada 2020^59^	Retrospective cohort	326	249.5 (8.4) ‐ days	Type 1–1500*	Not reported	Bladder injury 47 (14.42)	96 (29.45)	DIC 7 (2.82)
				Type 2–1500*		Ureteral damage 4 (1.23)		DIC 1 (2.27)
								DVT 1 (4.35)
				Type 3–2000*				DIC 4 (17.39)
				Type 4–2000*				
Nieto‐Calvache 2021	Retrospective case series	14	35 (35–36.1)*	1684.4 ± 680,7	0 (0–1)	Bladder injury 2 (14.3)	0	
Aditya Aryananda 2022	Retrospective cohort						Excluded	
‐ IIAL		36	35 (28–40)	1701 ± 813	2 (0–6)	Bladder injury 4 (11.8)		Vascular injury 1(2.8)
‐ OSCS		164	35 (28–39)*	1307 ± 743	1 (0–6)	Bladder injury 5 (3)	Excluded	ICU admission 3 (1.8)
								DIC 1 (0,6)
Nieto‐Calvache 2022	Prospective case series	64	36 (35–36)*	1500 (1100–2500)*	2 (1–2)	Bladder injury 3 (4.69)	0	
Palacios‐Jaraquemada 2023	Case series	13	Not reported	1500 (1400–2000)*	2 (0.5–2.5)*	Bladder injury 1 (7.7)	2 (15.4)	
Thi Pham 2023	Retrospective case series	65	35.4 ± 2.1	987 (677–1531)	Not reported	Bladder injury 3 (4.6)	4 (4.5)	Infection 2 (2.25)
						Insertion of JJ catheter 55(84.6)		
						Ureteral injury 1 (1.5)		
Vuong 2024	Retrospective cohort	217	35.25 ± 1.97	1000 (600–2000)*	Not reported	Bladder injury 9 (4.1)	24 (11.1)	Infection 22 (10,1)
						Insertion of JJ catheter 134 (61.8)		
Triple P								
Angileri 2017	Case series	37	35 + 2	2052.2	3.9 ± 2.4	Not reported	0	DVT 6 (16.2)
Tskhay 2017	Case series	17	34.3 ± 2.1	2423.5 ± 854	Not reported	Bladder injury 1 (5.9)	5 (29)	Not reported
Wei 2017	Retrospective cohort	45	36.2 ± 1.7	1078.7 ± 449.5	Not reported	Not reported	0	ICU admission 4 (8.8)
Abo‐Elroose 2019	Prospective case series	20	36.5 ± 0.8	1300 ± 300	Not reported	Bladder injury 1 (5)	1 (5)	Not reported
Pinas‐Carrillo 2019	Retrospective case series	50	24–37	2318 (400–7300)*	Not reported	Bladder injury 1 (2)	0	ICU admission 3 (6)
								DVT 3 (6)
Zhao 2022	Retrospective cohort	142	35 (34.1–35.9)*	1200 (687–1812)*	Not reported	Bladder injury 3 (2.1)	0	DVT 4 (2.8)
Other myometrial resection techniques								ICU admission 2 (1.4)
Barinov 2017	Retrospective case series							Not reported
Uterine sutures + vascular ligation (Group 1)		47	Not reported	Not reported	Not reported	Not reported	Group 1–26 (55)	
Uterine sutures + vascular ligation + intra‐uterine balloon (Group 2)		20					Group 2–2 (10)	
Uterine sutures + vascular ligation + intra‐uterine balloon + vaginal balloon (Group 3)		25					Group 3–0	
Kilicci 2017a	Prospective case series	11	Not reported	Not reported	2.1 ± 0,3	Bladder injury 1 (9)	2 (18)	Not reported
Kilicci 2017b	Retrospective cohort	22	35.3 ± 1.6	Not reported	1.1 ± 1.1	Bladder injury 1 (4.5)	Not reported	Not reported
Polat 2017	Retrospective cohort	Single incision −10	35.5 (29–36)*	Not reported	2 (0–4)	Not reported	Single incision ‐ 0	Not reported
		Double incision −12	35 (34–38)*		3,2 (0–13)		Double incision ‐ 1 (8.3)	
Shmakov 2018	Retrospective cohort							
Group 1 ‐ IIA ligation, myometrial resection		15	35.6 ± 1.5	2440 ± 1215	Not reported	Bladder wall resection 1 (6.67)	1 (6.7)	DVT 1 (6.7)
Group 2 ‐ CIA occlusion with vascular clamps; myometrial resection		18	34.8 ± 1.6	2186 ± 1353		Bladder wall resection 2 (11.11)	3 (16.7)	
Group 3 ‐ Uterine tourniquets; myometrial resection		21	34.3 ± 1.0	1295 ± 520.3		Bladder wall resection 1 (4.77)	1 (4.8)	
						Bladder injury 1(4.8)		
Zhao 2018	Retrospective case series	62	36	1377.3 ± 605.2	Not reported	Bladder injury 6 (9.7)	1 (1.6)	DVT 1 (1.6)
								ICU admission 1 (1.6)
								DIC 1 (1,6)
Peng 2019	Retrospective cohort	Group A ‐ 60	36.6 ± 1.2	2150 (800–6500)*	0 (0–15)*	Not reported	Group A ‐ 2/60 (3.3)	ICU admission 1 (1.7)
		Group B ‐ 64	35.8 ± 2.9	2800 (800–15 000)*	6 (0–31.5)*		Group B ‐ 14/64 (21.9)	ICU admission 19 (29.7)
Putra 2019	Retrospective case series	59	Not reported	594	Not reported	Bladder injury 1 (1.7)	0	Not reported
Zhou 2019	Retrospective case control				Not reported	Not reported		
‐ cases		58	36 ± 0.3	1215.5 ± 762.6			4 (6.9)	DIC 3 (5.2)
								DVT 1(1.72)
‐ controls		25	35.8 ± 0.3	1602 ± 862.5			3 (12)	DIC 3 (12)
Durukan 2020	Retrospective case series	23	36.7 ± 1.6	Not reported	3.0 ± 2.2	Urinary injuries 5 (21.7)	Excluded	Not reported
Kuznetsova 2020	Retrospective cohort		Not reported		Not reported		Not reported	Not reported
‐ bladder filled		22		2177.8 ± 114.9		Bladder injury 1 (4.5)		
						Ureteral injury 1 (4.5)		
‐ without filling the bladder		19		2545.7 ± 158.8		Bladder injury 6 (31.5)		
						Ureteral injury 1 (5.3)		
Üstünyurt 2020	Retrospective cohort	30	35.1 ± 2.4	877 ± 484	2.6 ± 3.1	Bladder injury 3 (10)	Excluded	ICU admission 2 (6.7)
Cirpan 2021	Retrospective case series	21	36.1 ± 1.51	Not reported	4.2 ± 2.0	None	2 (9.5)	Not reported
Ghaleb 2021	Prospective case series	62	35.5 ± 2.5	1700 (1200–2200)*	3.0 (2.0–4.0)*	Bladder injury 3 (4.8)	12 (19.4)	Not reported
Feng 2022	Retrospective case series	23	35.3 ± 2.5	5269.6 ± 2745.8	Not reported	Bladder injury 3 (13)	5 (22)	Not reported
Khallaf 2022	Case series	40	36.4 ± 1.4	Not reported	Not reported	Not reported	0	Not reported
Mousa 2023	Prospective case series	20	Not reported	1305 ± 361.6	1.3 ± 1.2	Bladder injury 1 (5)	2/20 (10)	Not reported
Paping 2024	Prospective cohort	38	36 (35–38)*	1500 (1000–2500)*	0 (0–4)*	Bladder injury 1 (2.6)	1 (2,6)	ICU admission 20 (52.6)
								DVT 1 (2,6)
Daggez 2024	Retrospective cohort	11	Not reported	1000*	0	0	Excluded	Not reported
Dilmy 2025	Retrospective cohort	81	36 (35–37)*	700 (500–1000)*		Not reported	Not reported	Not reported
Mousa 2025	Prospective cohort	12	35.17 ± 0.72	950 ± 233.55	1.58 ± 1.38	Bladder injury 1 (8.3)	1 (8.3)	Not reported
Total		2659	N/A	N/A	N/A	Bladder injury 119/1685 (7)	233/1801 (12.9)	
						Ureteral injury 9/1685 (0.53)		

*Note*: S1: upper bladder; S2: low bladder. T1: upper bladder; T2: parametrium; T3: low bladder; T4: low bladder with fibrosis.

Abbreviations: DIC, Disseminated intravascular coagulation; DVT, Deep vein thrombosis; IIAL, internal illiac artery ligation; N/A, not applicable; OSCS, one step conservative surgery.

* median.

**TABLE 2:** Surgical outcomes and intraoperative variables for placenta left in situ.

Author/year	Type of study	Number of patients	Gestational age at delivery (weeks)	Blood loss (mL)	Erythrocyte transfusion (units)	Urological interventions (%)	Hysterectomy (%)	Re‐operation (%)	Other outcomes (%)	Time to placental resolution
Sentilhes 2010	Retrospective case series	167	34.5 ± 4.75	NR	NR	NR	36 (21.6)	Delayed hysterectomy 18 (10.8)	Infection 47 (28.1)	Spontaneous 13.5 weeks (4 – 60)*
								Hysteroscopic resection / curettage 29/116 (25)	ICU admission 43 (25.7)	Hysteroscopic 20 weeks (2– 45) *
									Sepsis ‐ 8 (4.7)	
									DVT 3 (1.8)	
Amsalem 2011	Retrospective case series	10	35.2 ± 3.1	900 ± 754	2.0 ± 3.11	Bladder injury 1 (10)	4 (40)	Delayed hysterectomy 4 (40)	DIC 1 (10)	NR
									Bowel injury 1 (10)	
									Sepsis ‐ 1 (10)	
Breborowicz 2013	Retrospective case series	11	34.5	700 (300–1600)	NR	NR	5 (45.4)	Delayed hysterectomy 5 (45.4)	Sepsis ‐ 2 (18.8)	6–15 weeks
									DIC 3 (27,3)	
Fitzpatrick 2013	Retrospective cohort	32	NR	NR	7 (3–24)*	Vesicovaginal fistula 1 (3.2)	21 (65.6)	Delayed hysterectomy 21 (65.6)	ICU admission 22 (69)	NR
									Uterocutaneous fistula 1 (3.2)	
Kin Lo 2014	Retrospective case series	12	37 [33–38]*	1200 [500–3300]*	1 [0–10]*	NR	0	0	ICU admission 12 (100)	6.6 months (2–13)*
									Re‐admission 8 (67)	
Kutuk 2018	Retrospective cohort	15	NR	400 (250–2500)*	NR	0	1(7)	Delayed hysterectomy 1 (7)	Re‐admission 15 (100)	20.4 ± 6.9 weeks
Miyakoshi 2018	Retrospective case series	36	36 (28–38)*	1200 (300–6460)*	NR	NR	11 (30.6)	Hysteroscopic resection / curettage 4/25 (16)	NR	12.7 (1–72.8) weeks
Huang 2020	Retrospective case series	21	34.9 ± 3.3	854.7 ± 478.2	2.1 ± 2.0	NR	4 (19)	Dilation and curettage 4/17 (23,5)	ICU admission 2 (9.5)	4.69 ± 1.65 months
									Sepsis ‐ 3 (14.3)	
Lional 2020	Retrospective cohort	23	35.41	517.39	1.2	Bladder injury 4 (17.3)	9 (39.1)	Delayed hysterectomy 9 (39.1)	Re‐admission 15 (65.2)	13 months (6–22)*
						Ureteral injury 2 (8.6)			Sepsis ‐ 1 (4.3)	
									DIC 2 (8,6)	
Sentilhes 2021	Prospective cohort	86	NR	NR	NR	Bladder injury 3 (3.5)	19 (22.1)	Delayed hysterectomy 13 (15.1)	Re‐admission 24 (28.9)	
						Urinary fistula 1 (1.2)		Hysteroscopic resection / curettage 12/64 (18.8)	Bowel injury 1(1.2)	
Srinivasan 2021	Retrospective cohort	34	NR	NR	NR	Bladder injury 1 (2.9)	12 (35.3)	Delayed hysterectomy 2/24 (8.3)	ICU admission 11 (32.3)	Spontaneous resorption 181.1 ± 32.52 days
						Vesicovaginal fistula 1 (2.9)			DIC 4 (11.8)	Spontaneous expulsion 144.3 ± 56.67 days
van Beekhuizen 2021	Prospective/Retrospective cohort	48	NR	1500 (150–10 000)*	5 (0–108)*	Bladder injury 3 (6.25)	20 (41.7)	Hysteroscopic resection/curettage 8/28 (28,6)	ICU admission 24 (50)	29.1(21.40) weeks
								Re‐operation with myometrial resection 2/28 (7,1)		
Huang 2024		18	36*	744.44 ± 417.59	NR	NR	3 (16.7)	NR	NR	5.5 (2–8)* months
Paping 2024		10	37 (36–37)*	1600 (900–2800)*	2 (0–5)*	Bladder injury 1 (10)	6 (60)	NR	ICU admission 1 (10)	NR
Amro 2025	Retrospective cohort	29		1.100 (650–2.995)*		Intentional cystotomy 4 (14)	16 (55)	Curettage 3/13 (23)	Infection 3 (10)	17 weeks*
									ICU admission 5 (17)	
Total		552	NA	NA	NA	Bladder injury 20/287 (7)	167/552 (30.3)	135	Infection (including sepsis) 64	1/72 weeks
						Ureteral injury 2/287 (0.7)			DIC 10	
									DVT 3	
									ICU admission 120	

Abbreviations: DIC, Disseminated intravascular coagulopathy; DVT, Deep vein thrombosis; ICU, Intensive care unit; NR, Not reported.

* median.

**TABLE 3:** Recurrence of PAS after conservative surgery.

Author	Number of pregnancies	Recurrence of PAS (%)	Hysterectomy (%)
Myometrial resection			
Palacios‐Jaraquemada 2004	10	0	0
Palacios‐Jaraquemada 2021	202	0	0
Pinas‐Carrillo 2019	1	0	0
Zhao 2022	1	0	0
Khallaf 2022	3	1 (33)	0
Chen 2025	7	0	0
Total	224	1 (0.45)	0
Expectant management			
Sentilhes 2010	21	6	2
Sentilhes 2014	2	0	0
Chen 2025	7	1	1
Pineles 2022	4	0	0
Amro 2025	5	0	0
Total	39	7	3

### 3.1 Myometrial resection techniques

Myometrial resection involves en bloc excision of the placental implantation site together with the affected myometrium, followed by uterine reconstruction. Although multiple techniques have been described, all share common surgical principles: bladder dissection, control of aberrant neovascularization, devascularization of the placental bed, resection of the affected uterine wall, and reconstruction (Table 4).

**TABLE 4:** Surgical steps in uterine‐conserving techniques for placenta accreta spectrum.

OSCS	MOSCUS	Kasr Alainy	Triple P	Placenta in situ*
1‐ Dissection of the urinary bladder from the anterior uterine wall with vascular ligation of newly formed vessels (the Pelosi maneuver is used to evaluate the presence of vesicouterine fibrosis).	1‐ Dissection of the urinary bladder from the anterior uterine wall with vascular ligation of newly formed vessels.	1‐ Dissection of the urinary bladder from the anterior uterine wall with vascular ligation of newly formed vessels.	1 ‐ Placement of occlusion balloon catheters in the anterior division of the internal iliac artery.	Midline periumbilical abdominal incision.
2 ‐ Hysterotomy, immediately above the abnormal area (Ward's maneuver).	2 ‐ Incision of the lower uterine segment at the upper margin of the placenta or cutting through the placenta in unavoidable cases.	2 ‐ Uterine tourniquet with Foley catheter (18 Fr).	2 ‐ Peri‐operative placental localization and transverse uterine incision above the upper border of the placenta, with abdominal ultrasound performed during surgery.	Hysterotomy away from the placenta (++ fundal).
3 ‐ Uterus exteriorization and control of colpo‐uterine pedicles with 3 simple “X stitches” involving 3 cm of healthy myometrium.	3‐ A tourniquet is put into the para‐cervical area (using a Foley 12‐F catheter), and bilateral uterine artery ligation (using Chromic 1/0) is performed.	3‐ Transverse uterine incision just above the PAS upper border.	3 ‐ Pelvic devascularization: after the delivery of the neonate by inflating the occlusive balloon catheters.	Attempt to gently remove the placenta only in cases of unconvincing findings of PAS.
4 ‐ Resection of the abnormal myometrium along with the placenta; hemostatic sutures of the placental bed surface as needed.	4 ‐ Resection of all invaded myometrial portions, manual removal of the whole placenta, or left partial placenta in situ, hemostasis of local bleeding vessels.	4‐ Placental bed devascularisation (uterine arteries ligation, high and low; cervical vessels ligation, anterior and posterior wall).	4 ‐ Placental non‐separation with myometrial excision and reconstruction of the uterine wall; application of three, interrupted “Box Stitches,” two at the angles and one at the center, followed by a continuous suture.	Placenta left in situ, the umbilical cord is cut, and the hysterotomy is closed with no uterotonics.
5 ‐ Uterine reconstruction, two layers. The first with “U” stitches joining the borders of the hysterotomy. The second is an unlocked, simple continuous suture	5 ‐ Suture of both edges of the uterine incision and hemostatic sutures of the placental bed surface.	5‐ Enbloc resection of the invaded segment of the uterine wall, along with the invading placenta; hemostatic sutures of the placental bed surface as needed.	5 ‐ With bladder involvement, the placenta is partially left in situ. As much of the placenta as possible is removed, and multiple hemostatic sutures are applied to this area.	Evaluate intra‐operatively for any abnormal bleeding.
	6 ‐ Transverse B‐Lynch suture, using atraumatic number 1 chromic catgut, after releasing the tourniquet.	6 ‐ Myometrial reconstruction in 2 layers (continuous polyglactin 1–0 sutures).	6‐ With PAS on the cervix, the use of the Bakri balloon inflated with approximately 150 ml of warm saline, and retained by two vertical compression sutures (vertical sandwich technique) is recommended.	Post‐partum broad‐spectrum antibiotics (5 day course of gentamicin 5 mg/kg IV every 24 h and clindamycin 900‐mg IV every 8 h).
	7 ‐ Uterine reconstruction with 1 layer continuous suture.			Long‐term follow‐up for placental reabsorption (with possible hysteroscopy or dilation and curettage).
	8 ‐ Bladder repair if necessary.			

Abbreviations: MOSCUS, modified one step conservative surgery; PAS, placenta accreta spectrum; OSCS, one step conservative surgery.

* according to Pineles et al.^49^

#### 3.1.1 One‐step conservative surgery

**FIGURE 1:**
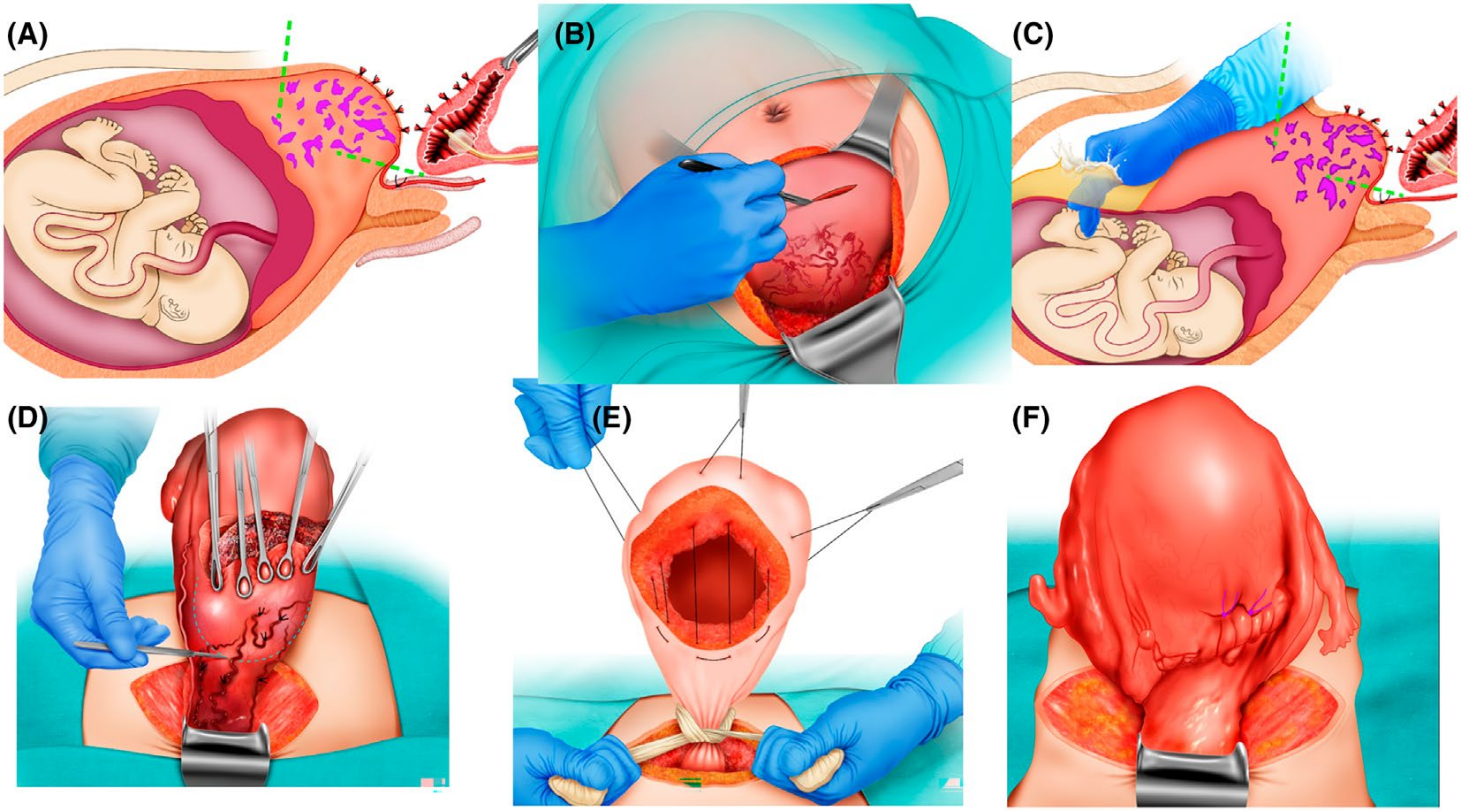
One‐step conservative surgery for placenta accreta spectrum. (A) Placenta accreta located in the lower uterine segment (LUS) with a bulging area (enclosed by green dotted lines) becomes evident after bladder dissection and ligation of the vesicouterine pedicles connecting the bladder to the LUS. In the anterior bladder wall, an ascending colpouterine artery toward the LUS can be observed. This vessel has been ligated. (B) After surgical staging, a hysterotomy is performed approximately 1 cm above the bulging and hypervascularized area, through healthy myometrium. (C) Fetal extraction is carried out while avoiding placental rupture, using the Ward maneuver, in which the surgeon, after performing the segmental incision and encountering placental tissue, introduces the hand between the placenta (in its usually implanted portion) and the myometrium to produce a controlled separation (abruptio) until reaching the nearest placental edge, then ruptures the membranes and exteriorizes the detached placental border to deliver the fetus. (D) The uterus is exteriorized, and bleeding from the hysterotomy edges is controlled with ring forceps. The colpouterine vessels have been ligated at the lowest part of the uterine segment, both in the midline and bilaterally. En bloc resection of the abnormal myometrium and adherent placenta (indicated by the green dotted line) is then performed. (E) Hysterorrhaphy begins with a first layer of interrupted “U”‐shaped sutures. Steps D and E may be performed with a uterine tourniquet applied caudally over healthy myometrium to reduce blood loss. (F) The second layer of hysterorrhaphy is completed with a continuous, unlocked, inverting suture.

Originally described in 1998, OSCS requires adequate mobilization of the bladder and sufficient residual healthy myometrium—generally at least 2 cm above the cervix and less than 50% circumferential involvement—to permit uterine reconstruction^13^, ^18^ (Figure 1). Furthermore, a topographic evaluation of the placenta should be performed, with special attention to cases of lower lateral and lower anterior uterine wall involvement with insufficient residual healthy myometrium, where OSCS may not be feasible.^19^ The abdominal incision may be a modified Pfannenstiel or a midline incision. Digital opening of the parametrial space, close to the round ligament of the uterus, allows for an objective evaluation of the lateral uterine wall and determines the limits of the anterior wall injury. After this, the separation of the uterus from the bladder is performed. The Pelosi maneuver, a method used to evaluate the presence of vesicouterine fibrosis and the difficulty in separating the two organs, is performed. Following this, the surgeon must identify and ligate each of the neo‐vessels that run between the bladder and uterus (vesicouterine pedicles); for this, vessels must be tied with sutures or ligated with advanced surgical instruments. Only after this is the hysterotomy performed, in the upper segment, immediately above the myometrial bulging area, using the Ward's maneuver to avoid placing the incision too high, which could make myometrial reconstruction difficult.^20^ After fetal extraction, while the surgical assistant applies traction on the uterus anteriorly and cephalically, the surgeon assesses the healthy caudal myometrium and places three hemostatic stitches to ligate the colpouterine arterial pedicles. These vessels form an anastomotic pedicle, connecting branches of the internal pudendal artery with the uterine arteries. In cases antenatally predicted as severe, a balloon may be placed intraoperatively at the level of the infrarenal aorta, without the need for fluoroscopic guidance. Alternatively, aortic control can be achieved using manual compression, a vascular clamp, or ligature.^21^ After completing the previous steps, the surgeon cuts the abnormal myometrium from the edges of the hysterotomy and then removes the placenta and the area of abnormal uterine wall en bloc. The uterus should then be repaired in two layers: first, a mattress suture, and then a continuous suture with one polyglactin acid.^18^

This technique was later modified by Thi Pham et al. (MOSCUS technique),^22^, ^23^ incorporating a transverse B‐Lynch compression suture (using atraumatic number 1 chromic catgut), a lower uterine segment tourniquet, and a hysterotomy closure with a single layer of suture. Some other procedures have been reported in the literature, with small modifications.^24^, ^25^, ^26^, ^27^, ^28^, ^29^, ^30^, ^31^, ^32^, ^33^, ^34^, ^35^, ^36^, ^37^, ^38^, ^39^, ^40^, ^41^

### 3.2 Kasr Alainy technique

**FIGURE 2:**
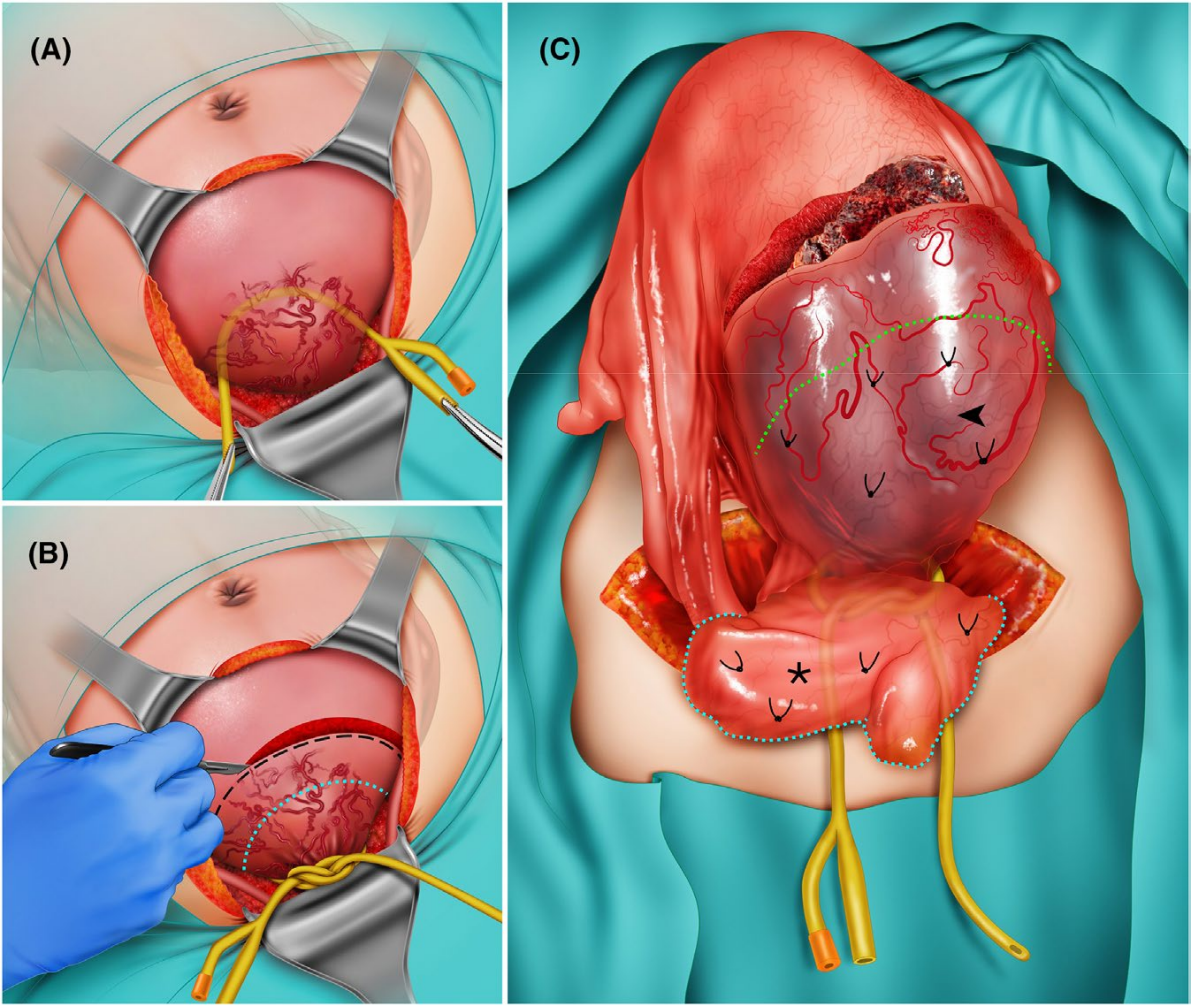
Kasr Alainy technique for the management of placenta accreta spectrum. Similar steps to the one‐step conservative surgery are already depicted in Figure 1. A Foley catheter is passed through an opening in the broad ligaments (A) while bladder dissection is performed. The lower uterine segment is compressed by tightening the tourniquet, and the fetus is delivered through a transverse incision made at the edge of the abnormal bulging area (B). The bladder is included within the tourniquet loop (blue dotted line in B shows the upper border of the bladder over the bulging area of the uterus) so that the retrovesical dissection (including vesicouterine pedicles control) can be completed without additional bleeding. After fetal delivery, the uterus is exteriorized, and bladder dissection is completed while bleeding remains controlled by the uterine tourniquet (C). The asterisk in panel C indicates the posterior surface of the bladder, which has been dissected from the anterior surface of the uterine segment (arrowhead), with the vesicouterine pedicles being ligated one by one. The blue dotted line marks the bladder edge that, before dissection, was adherent to the uterine segment (at the level of the green dotted line). After achieving hemostasis on the posterior bladder surface, the uterine arteries are ligated, the tourniquet is loosened, the bladder is released, and the tourniquet is repositioned to include only the uterus, allowing completion of the en bloc resection of the abnormal area or hysterectomy. Any uterine bleeding sites are secured with hemostatic sutures before final loosening of the tourniquet.

The Kasr Alainy surgery (Figure 2) was first described in 2023 (Table 4). The procedure shares several steps with OSCS, but includes a uterine tourniquet with a Foley catheter (18 Fr), placental bed devascularization with uterine artery ligation (both low and high uterine ligation), and ligation of the anterior and posterior cervical vessels; lower uterine artery ligation should be positioned higher than the ureteric tunnel to avoid iatrogenic lesions. The reconstruction of the hysterotomy is performed by two layers of continuous running sutures, using an absorbable suture. In cases of cervical PAS, the bladder is reflected caudally to the level of the anterior vaginal fornix, and a U‐shaped anterior cervical suture is performed, encircling the distal cervix/internal os and compressing the placental bed to prevent bleeding from the distal cervical stroma.^40^ To reduce plastering of the newly reconstructed lower uterine segment to the denuded surface of the bladder back and anterior uterine wall, it is possible to plicate the lateral vesical pads of fat into the depth of the uterovesical pouch and fix it to the cervix with two Vicryl 2/0 sutures.

### 3.3 Triple P

**FIGURE 3:**
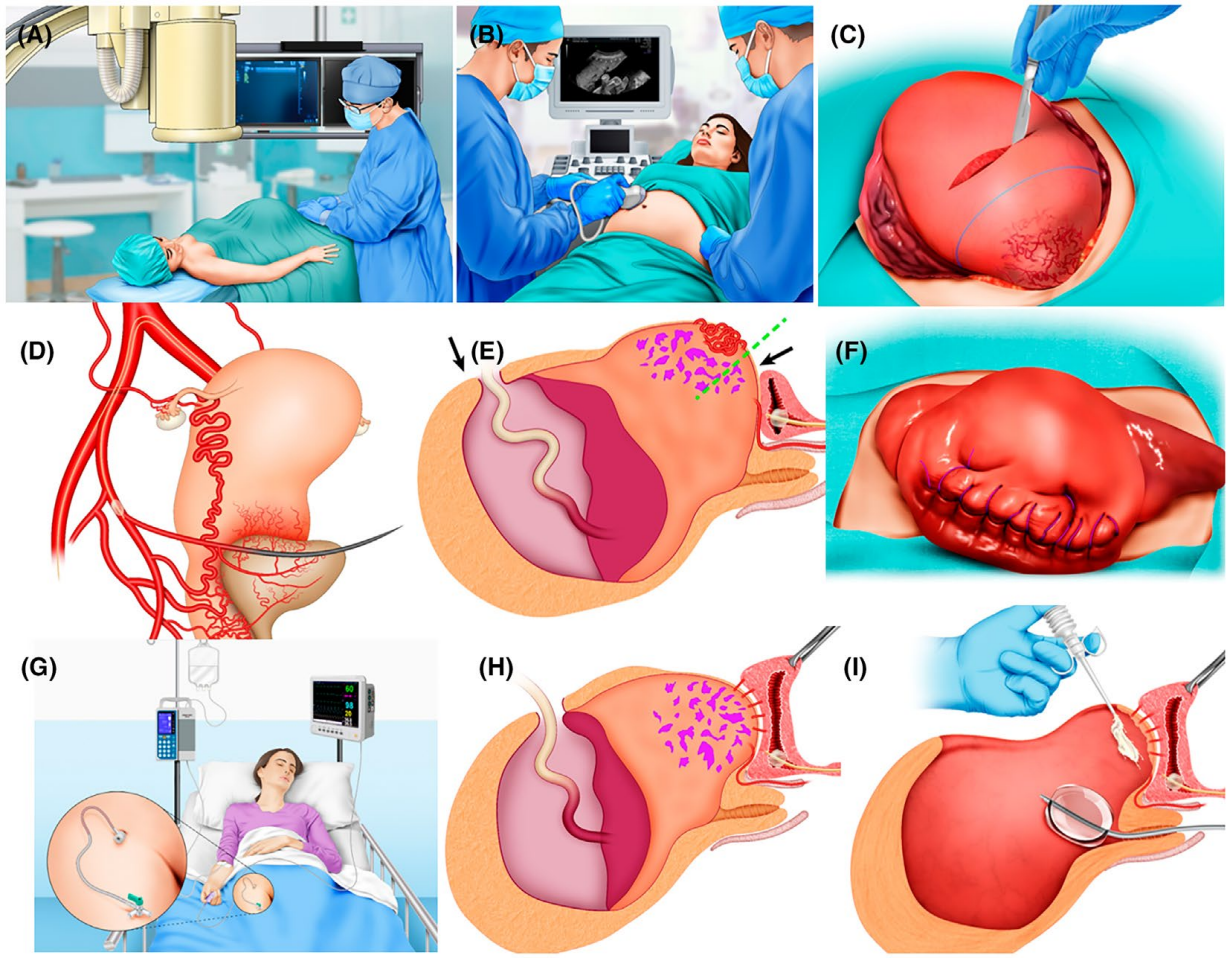
Triple‐P procedure for placenta accreta spectrum. (A) Preoperative placement of occlusion balloon catheters in the anterior division of the internal iliac arteries. (B) Preoperative mapping of the placenta to select the hysterotomy site away from the placental edge. (C) The blue line indicates the placental margin identified by prenatal ultrasound. The transverse hysterotomy is performed two finger breadths above this placental border. (D) Inflation of endovascular balloons in the anterior division of the internal iliac arteries. (E) Placental non‐separation with excision of the involved myometrium in lesions located above the vesicouterine peritoneal reflection. (F) Reconstruction of the uterine wall ‐ three, interrupted “Box Stitches,” two at the angles and one at the center for approximation of the upper and lower margins of the myometrial defect, followed by a continuous suture applied to the edges of the myometrial incision. (G) Femoral introducers are left in place until the day after surgery to allow interventional radiology access if required. (H,I) In lesions involving the lower uterine segment (H, “bladder involvement”), the placenta is partially left in situ. As much placental tissue as possible is removed (I), and multiple hemostatic sutures are placed in this area. When necessary, topical thrombin is applied over the bleeding surface of the uterine segment adherent to the bladder, and a Bakri balloon may be inflated with approximately 150 mL of warm saline and positioned over the bleeding area before uterine closure, especially in cases of cervical PAS (I).

The triple P procedure, originally described in 2012 (Figure 3) comprises Perioperative placental localization, Pelvic devascularization, and Placental non‐separation with myometrial excision and reconstruction of the uterine wall (Table 4).^42^ The procedure involves:

1. Perioperative placental localization and delivery of the fetus via transverse uterine incision above the upper border of the placenta, with abdominal ultrasound performed in the operating room. The myometrial incision is made two finger breadths above the initial ultrasound measurement.
2. Before surgery, occlusion balloon catheters are placed in the anterior divisions of the internal iliac arteries in the interventional radiology suite. If interventional radiology facilities are unavailable, ligation of the uterine arteries may be performed. Pelvic devascularization is carried out immediately after the delivery of the neonate by inflating the pre‐positioned occlusive balloon catheter.
3. Placental non‐separation with myometrial excision and reconstruction of the uterine wall. The entire anterior wall of the myometrium is excised, and the lateral border of the excision is extended to the angles of the uterine incision.

Some modifications have been described, such as surgery performed with uterine artery ligation, uterine tourniquet, or catheters in the abdominal aorta.^43^, ^44^, ^45^

In cases of bladder involvement, the placenta is partially left in situ. Once the myometrium is excised with the bulk of the placenta, the lower lip of the incision can be everted to expose the line of adherence of the trophoblastic tissue into the posterior wall of the bladder. As much placenta as possible is removed piecemeal from the inferior edge of the uterine incision, and multiple hemostatic sutures are applied to this area with further application of local hemostatic agents and of constant pressure with a warm saline pack. Hydrostatic balloon tamponade is also used for complex cases involving the uterine cervix, inflated with approximately 150 mL of warm saline, and retained by two vertical compression sutures (vertical sandwich technique).^46^

Balloon catheters are deflated 2–4 h post‐delivery, once bleeding has stopped. However, the femoral sheath is left in place until the day after surgery to allow for interventional radiology access if required.

### 3.4 Leaving the placenta in situ

**FIGURE 4:**
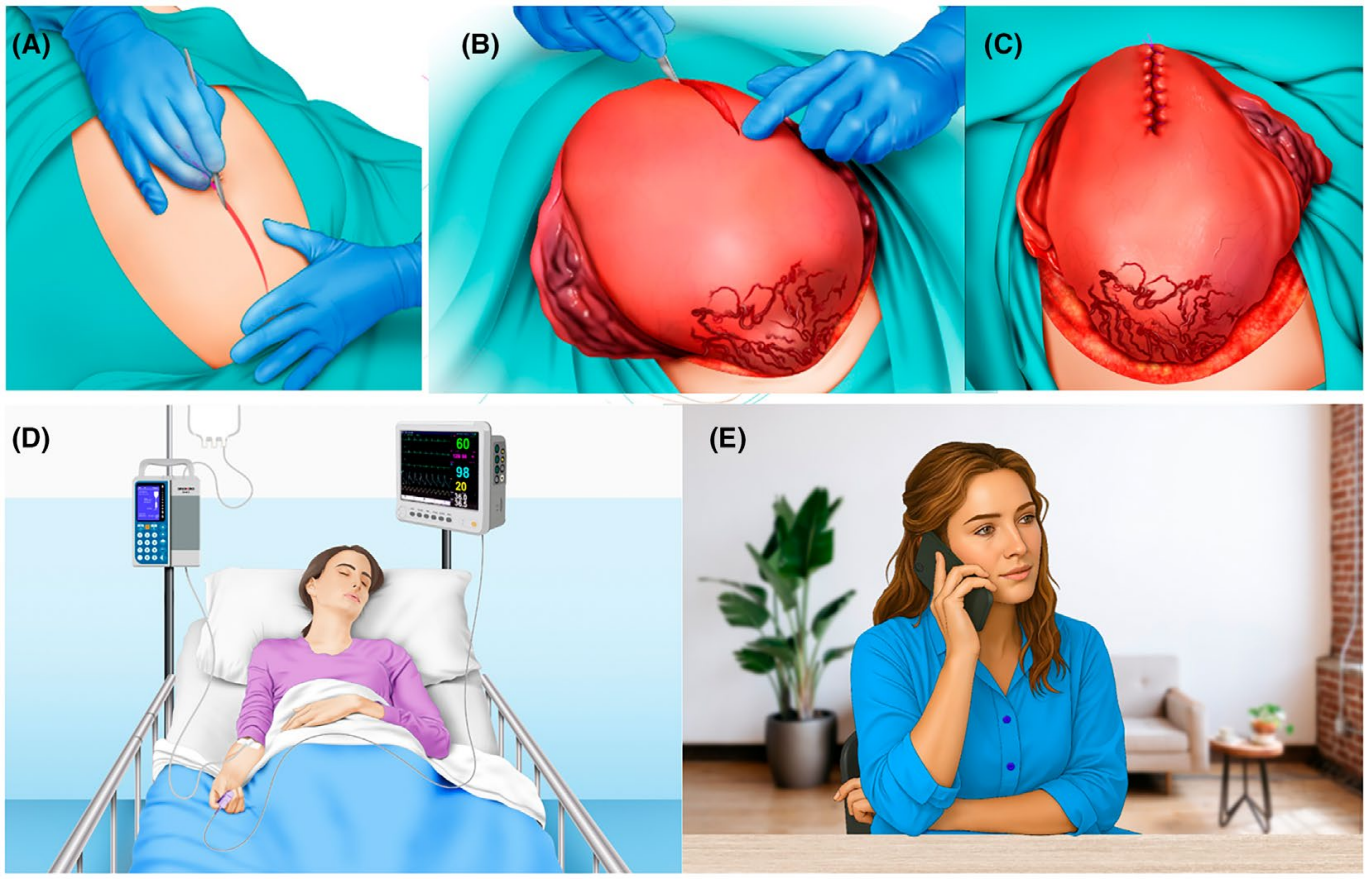
Leaving the placenta in situ for the management of placenta accreta spectrum (according to Pineles et al.) (49). (A) Midline periumbilical abdominal incision. (B) Hysterotomy is performed away from the placental margin, in most cases, in the fundus. (C) The placenta is left in situ; the umbilical cord is cut, and the hysterotomy is closed. (D) Administration of broad‐spectrum antibiotics in the postpartum period. (E) Long‐term follow‐up to monitor placental resorption.

The approach of leaving the placenta in situ (Figure 4) for the management of PAS was first described in the literature in 1933 in Italy and later in 1951 in the United States.^47^, ^48^ The method relies on spontaneous necrosis and gradual resorption of the retained placenta following delivery, as uterine blood flow decreases (Table 4). Rigorous selection criteria must be applied to ensure adequate candidate selection for this procedure.^49^ An attempt to separate the placenta may be made in cases where high‐grade PAS is discarded. In this approach, the placenta adhering to the myometrium is left in situ, either totally or after removing the non‐adherent part. Additional procedures, such as pelvic devascularization (including pelvic arterial embolization, vessel ligation, or uterine compression sutures), preoperative ureteric stent placement, and balloon catheter occlusion, can be performed at the surgeon's discretion. Prophylactic antibiotics are regularly prescribed after surgery.^14^, ^49^ The use of methotrexate to accelerate placental resorption remains controversial, as the syncytiotrophoblast in a mature placenta lacks proliferative activity, making the drug ineffective and potentially harmful. Both FIGO and IS‐PAS guidelines recommend against its use, especially in breastfeeding women.^9^, ^14^ According to Pineles et al.^49^ discharge is possible after 7–10 days, without significant bleeding or signs of sepsis.

Patients with the placenta in situ require strict and prolonged follow‐up. FIGO recommends weekly outpatient evaluations for the first 2 months, followed by monthly visits until placental resorption is confirmed.^14^ Follow‐up should assess signs of infection and vaginal bleeding and should include clinical examination, pelvic ultrasound, and laboratory tests. The distance from the hospital should also be considered, as secondary complications may require immediate assessment at a 24/7 hospital. The placenta can either be reabsorbed, expelled, or removed by hysteroscopic surgery or curettage.^50^, ^51^, ^52^ Vaginal discharge may persist for months after delivery.^49^ Importantly, histopathological evaluation is not possible in cases when the placenta is in situ.

### 3.5 Outcomes assessed

The primary analysis included 35 studies on myometrial resection procedures (2659 patients)^15^, ^18^, ^22^, ^23^, ^24^, ^25^, ^26^, ^27^, ^28^, ^29^, ^30^, ^31^, ^32^, ^33^, ^35^, ^36^, ^37^, ^38^, ^39^, ^40^, ^43^, ^44^, ^45^, ^53^, ^54^, ^55^, ^56^, ^57^, ^58^, ^59^, ^60^, ^61^, ^62^ and 15 studies with the placenta in situ (552 patients).^15^, ^63^, ^64^, ^65^, ^66^, ^67^, ^68^, ^69^, ^70^, ^71^, ^72^, ^73^, ^74^, ^75^, ^76^ The main characteristics of the studies included are depicted in Tables 1, 2, 3. Hysterectomy was ultimately required in 12.9% of myometrial resection cases and 30.3% of expectant management cases. Bladder injury occurred in approximately 7% of cases in both groups, while ureteric injury was rare (< one %). Infectious morbidity and delayed reoperations were substantially more frequent when the placenta was left in situ. The time to placental resorption with placenta in situ was variably defined, with a maximum of approximately 72 weeks after delivery. Regarding fertility outcomes, 224 pregnancies were reported after myometrial resection, with a single recurrence of PAS. In contrast, 39 pregnancies were reported following expectant management, with seven PAS recurrences and three hysterectomies.

### 3.6 Comments

#### 3.6.1 Main findings and clinical interpretation

This expert review highlights the fundamental differences between myometrial resection techniques and leaving the placenta in situ for PAS management. PAS is a spectrum disorder, and no single strategy is universally applicable. Management should therefore be individualized, based on placental topography, surgical expertise, institutional resources, and patient preferences. Several procedures have been performed, yielding excellent uterine preservation rates and morbidity comparable to that of cesarean hysterectomy, as indicated by this study. Consequently, in referral centres, both cesarean hysterectomy and conservative approaches should be available and clearly discussed with patients, allowing each case to be managed in a personalized and informed manner. A critical principle across all conservative approaches is readiness to abandon uterus‐preserving strategies and proceed to hysterectomy when conservative management becomes unsafe, such as in cases of uncontrolled hemorrhage or unfavorable placental anatomy. In such cases, a standardized approach to hysterectomy should also be available.^12^

The OSCS has already been performed in many different countries, including most of Latin America (Argentina, Peru, Colombia, Brazil, Bolivia, Guatemala, Nicaragua, Panama, Mexico, Ecuador, Venezuela), the United States of America, Indonesia, China, Egypt, Morocco, Algeria, Kazakhstan, Russia, Switzerland, Sweden, Lithuania, Poland, Spain, England, Ireland and Portugal, both after training at a hospital with experience in the surgery or a virtual training program.^77^ Tele‐education and telepresence during PAS surgery may help implement OSCS in selected cases, thereby expanding access to these surgical procedures. All myometrial resection procedures follow the same basic principles, including dissection of the bladder from the anterior uterine wall, vascular control techniques, and myometrial resection. Many of the surgical steps performed are also required in a hysterectomy, which may shorten the learning curve for experienced surgeons. Additionally, if a hysterectomy is needed, many of the essential steps may already have been completed. However, some differences exist between the procedures described, as shown in Table 4. An essential difference between the Triple P and other myometrial resection procedures is the leaving of the placenta in situ (partially) in cases of PAS with involvement of the lower uterine segment, below the vesicouterine peritoneum reflection, being a hybrid approach between conservative treatment and leaving the placenta in situ in PAS. By examining our data using standardized techniques, including OSCS, MOSCUS, and the Kasr Alainy procedure, the studies included report similar mean/median blood losses, all of which are inferior to 2000 mL. With the Triple P procedure, although data are more heterogeneous, reports of blood loss are also mostly around or below 2000 mL. These procedures are associated with a low rate of urological complications as well as an even lower rate of other complications (vascular, infectious, or other organ injuries), and the uterus may be preserved in more than 80% of cases.

Leaving the placenta in situ may also be successful in around 70% of cases. Furthermore, it may be particularly useful in cases of unexpected PAS with an inexperienced team, especially in resource‐constrained settings, where assembling a multidisciplinary team within a short period is impossible.^78^ It is essential to acknowledge that these patients are prone to a higher risk for delayed complications, including post‐operative bleeding with intravascular disseminated coagulation and sepsis, potentially ending up in delayed hysterectomy. Cases of secondary hysterectomy due to severe bleeding were described more than 2 months after the cesarean section, meaning that patients may continue to be at risk of severe bleeding for several months after delivery.^66^ As a consequence, these patients should be thoroughly informed about the essential long‐term follow‐up period, with monitoring until complete reabsorption of the placenta or hysteroscopic removal of placental fragments. In the study by Sentilhes et al., the placenta could take 4–60 weeks to complete reabsorption (median, 13.5 weeks) or undergo hysteroscopic removal (median, 20 weeks). The median period until delayed hysterectomy was 22 weeks (interquartile range, 9–45).^63^ Lastly, women should be informed about the possibility of vaginal discharge (up to 17 weeks after delivery in some studies), pelvic pain (up to 25 weeks), and unexpected bleeding for weeks.^49^ As an example, in the study by Amro et al., nine of 16 (56%) hysterectomies were performed as a result of patient request, due to inability to continue frequent follow‐up, anxiety, uterine cramping, or foul‐smelling lochia.^76^ Recently, several authors have recommended expectant management for PAS to minimize the risks of severe maternal morbidity and mortality associated with a peripartum hysterectomy, even in countries where PAS was traditionally managed with a peripartum hysterectomy, such as the United States of America.^76^, ^79^

Furthermore, when adequately performed, conservative surgeries may be an important tool in low‐resource settings and in countries where, for cultural and religious beliefs, uterine conservation may be considered essential. Particularly, the OSCS and the Kasr Alainy technique, which are performed without the need for expensive resources (Triple P and leaving the placenta in situ may also be performed without interventional radiology techniques) and avoid extensive retroperitoneal dissections, may be interesting in settings where resources may pose extra difficulties. Additionally, these data, along with those from our previous meta‐analysis^17^ regarding intra‐operative blood losses, intra‐operative complications, and intensive care unit admissions, these procedures are particularly interesting in low‐resource settings, where resources are more limited, and blood banks may face particular difficulties.

Regarding fertility outcomes, we found 224 pregnancies after myometrial resection procedures, with one case of PAS recurrence (0.5%) and 39 pregnancies with expectant management, with seven instances of recurrence (18%) and three hysterectomies. Our data is in accordance with the results presented by Baldwin et al.,^80^ who reported in their population‐based study, rates of recurrence of PAS of 4.7% (95% CI 3.0–6.5%) for the second, 7.6% (95% CI 2.8–12.3%) for the third pregnancy after PAS in the preceding birth. Women should be counseled that fertility preservation is not guaranteed and that future pregnancies remain high‐risk.

Some limitations of this study should be noted. First, we note heterogeneity in PAS diagnosis. It is possible that some cases of uterine dehiscence were misclassified as PAS, biasing the results toward better outcomes; also, by leaving the placenta in situ, pathological confirmation of the suspicion of PAS is not possible. Most studies were retrospective; these studies are associated with a higher risk of bias in estimating blood loss and transfusion rates. The lack of randomized controlled studies in PAS limits the conclusions of this study. As a spectrum disease, it is important to acknowledge that each situation is unique. We understand that an RCT is feasible, requiring a multicenter study with a strict protocol and precise, clearly defined outcomes. Furthermore, we believe there is now sufficient evidence for conservative surgery in PAS, thereby clearly establishing its role in the treatment of this entity. There is also the possibility of confounding, with the most favorable cases being those in which the placenta is left in situ or conservative surgery is performed. Further research is needed to establish and validate criteria for selecting candidate patients for either procedure. Blood loss when leaving the placenta in situ should be considered, both in cases of cesarean section and secondary intervention when needed, a consideration that was not consistently described in the studies.

## 4 CONCLUSION AND IMPLICATIONS

Conservative surgical management of PAS is feasible and has been successfully implemented in specialized centers worldwide. Clear differentiation between myometrial resection techniques and leaving the placenta in situ is essential to avoid misinterpretation of outcomes and to guide appropriate patient counseling. Uterus‐preserving strategies may be considered in selected cases, provided that multidisciplinary expertise and readiness for hysterectomy are ensured. In these cases, the patient's perspective is crucial, and a holistic, shared decision‐making process is essential. Careful counseling is paramount, reinforcing the need for prolonged follow‐up, especially in cases where the placenta is left in situ. Although fertility preservation may not be the primary concern in all cases, the low recurrence rates of PAS following both conservative approaches are reassuring. Our findings suggest that a paradigm shift in PAS management may be emerging—favoring conservative procedures when clinically appropriate and feasible. Ultimately, PAS surgery—whether conservative or radical—should be performed in referral centers by teams with advanced surgical training and institutional support.

## AUTHOR CONTRIBUTIONS

PVP: conceptualization, data curation, formal analysis, methodology, supervision, writing – original draft. AJN: conceptualization, data curation, supervision, writing – review and editing. RJV: data curation, formal analysis, methodology, writing – review and editing. AM: conceptualization, data curation, supervision, writing – review and editing. HS: conceptualization, data curation, supervision, writing – review and editing. RAA: conceptualization, data curation, supervision, writing – review and editing. JMPJ: conceptualization, supervision, writing – review and editing.

## FUNDING INFORMATION

This research did not receive any specific grant from funding agencies in the public, commercial, or not‐for‐profit sectors. There was no funding for this study.

## CONFLICT OF INTEREST STATEMENT

The authors declare that they have no conflict of interest. The authors declare that they have no competing interests.

## Data Availability

Data sharing not applicable to this article as no datasets were generated or analysed during the current study.
